# Thiazole Orange Fluoresces Freely: No Rigid Environment Required

**DOI:** 10.1021/acs.jpclett.6c02059

**Published:** 2026-07-18

**Authors:** Kim Greis, Thomas Toft Lindkvist, Michael Schreivogel, Iden Djavani-Tabrizi, Franco Molina, Steen Brøndsted Nielsen, Renato Zenobi

**Affiliations:** † Laboratory of Organic Chemistry, Department of Chemistry and Applied Biosciences, 27219 ETH Zürich, CH-8093 Zürich, Switzerland; ‡ Department of Physics and Astronomy, 1006 Aarhus University, DK-8000 Aarhus, Denmark

## Abstract

Twist motion is a common relaxation pathway in many fluorescent photoswitches, including the green fluorescent protein chromophore, thioflavins, and thiazole orange (TO); but the associated excited-state dynamics are highly sensitive to the local environment. In this work, we have studied the cationic dye TO that is virtually nonemissive in water but displays strong turn-on fluorescence upon DNA binding. We demonstrate that fluorescence is an intrinsic property of TO that does not require a rigid environment, as isolated TO cations in vacuo exhibit strong fluorescence with an unexpectedly long 11 ns lifetime, 3 orders of magnitude larger than its picosecond lifetime in water. Cryogenic fluorescence ion spectroscopy reveals that absorption and fluorescence spectra of isolated TO resemble those of the DNA-bound dye, which indicates that fluorescence from the gas-phase ions occurs from a near-planar geometry. We show that in the gas phase there is an intrinsic barrier on the excited-state potential energy surface along the twist coordinate preventing deexcitation through a conical intersection with the ground-state surface, as observed in water. Quantum-chemical calculations of Franck–Condon spectra and potential energy surfaces support our interpretation and challenge the prevailing assumption that a rigid environment is needed to turn on fluorescence in TO.

## Graphical abstract

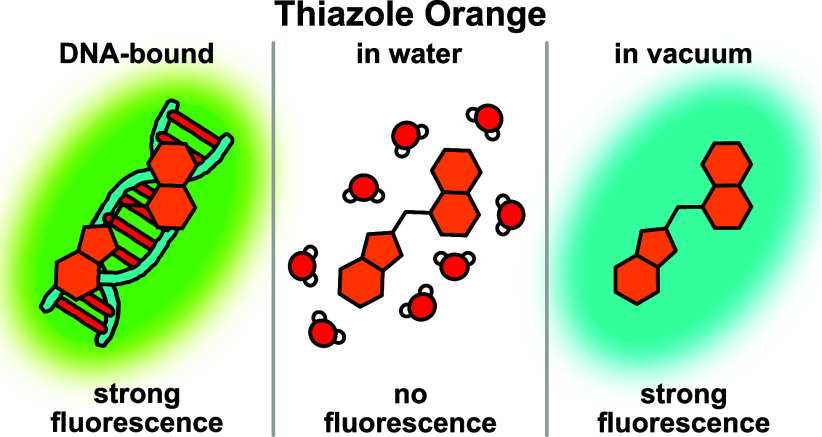

Thiazole orange (TO) is a cationic asymmetric monomethine cyanine dye (Scheme 1) and is used extensively to stain DNA and RNA with high contrast. In solution, TO displays a near-zero fluorescence quantum yield of 0.0002, which increases to ca. 0.1 upon complexation with DNA.^1^ Time-resolved spectroscopy experiments in water^2^ and methanol^3^ showed rapid quenching of fluorescence ascribed to twisting to a conical intersection (CI), through which it returns to the ground state within a few picoseconds. When the dye intercalates with DNA or RNA, this twist motion is sterically hindered, and the fluorescent geometry is stabilized. Correspondingly, the excited-state lifetime of TO-DNA complexes increases to a few nanoseconds.^4,5^

**Scheme 1:**
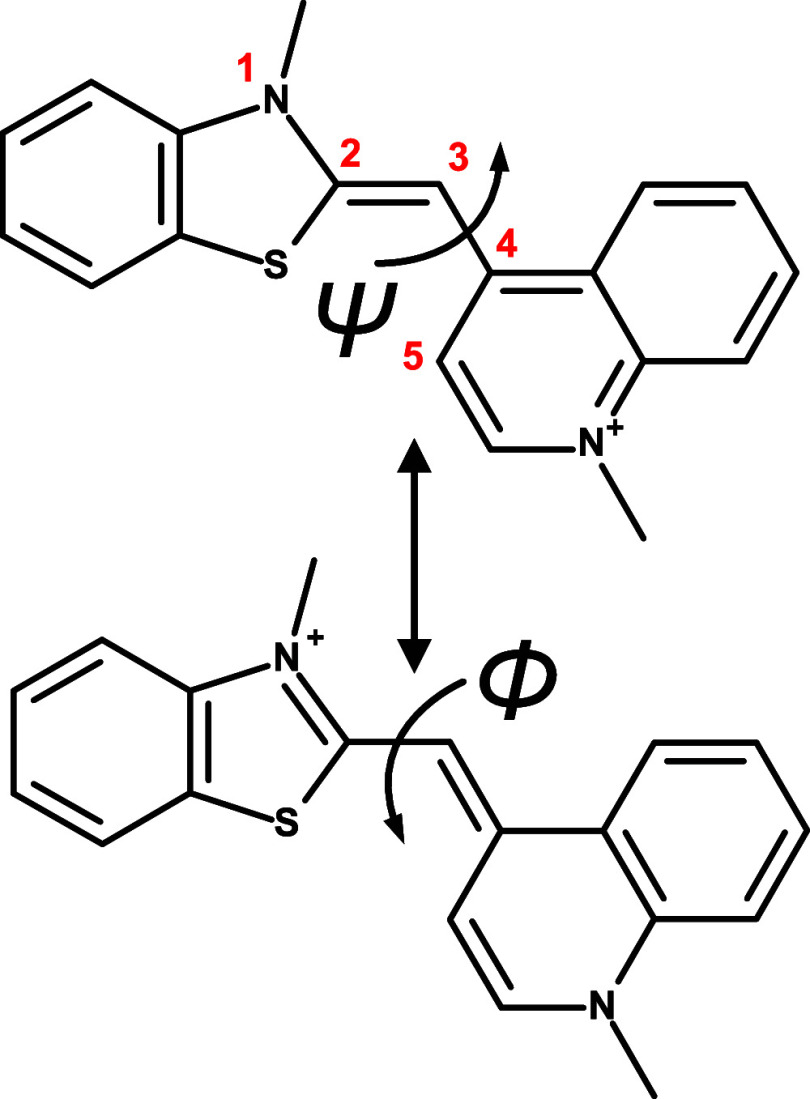
Two Dominant Resonance Forms of TO in Its Lowest-Energy *trans* Structure^a^

a We define the two dihedral (D) angles *ϕ* = *D*(1,2,3,4) and *ψ* = *D*(2,3,4,5).

To reveal more about the intrinsic properties of TO and its fluorescence-quenching mechanism, particularly the nature of the excited-state twisting motion, we have studied TO in the gas phase, free from solvent, using custom-built setups for fluorescence ion spectroscopy. Room-temperature (RT) time-correlated single-photon counting (TCSPC) experiments were conducted at ETH Zürich to measure the excited-state lifetimes in the gas phase as well as in solution. Complementary low-temperature gas-phase absorption and emission spectra were measured with LUNA2 (LUminescence iNstrument in Aarhus 2). Both experiments^6,7^ employed electrospray ionization (ESI) to transfer isolated TO into an ion trap modified to allow for laser excitation and fluorescence collection (see the Supporting Information (SI) for details). ESI leads to the formation of TO monomers (Figure S1), hereby avoiding complications caused by dimer formation as observed in solution phase. Additionally, we conducted Franck–Condon (FC) simulations of absorption and emission spectra together with calculations of the ground- and excited-state potential energy surfaces (PESs) with time-dependent density-functional theory (TD-DFT) and state-averaged complete active space self-consistent field (SA-CASSCF) methods.

Evident from Figure 1A, fluorescence from TO in water quickly decays with an excited-state lifetime below 25 ps. The decay is multiexponential, and longer lifetime components were previously ascribed to self-stabilizing dimers and aggregates.^2,8^ The shortest lifetime component in solution is consistent with a very low or nonexistent barrier to excited-state deactivation as argued previously.^3^ Quenching of fluorescence via the twist motion is slowed in viscous solvents,^4^ as seen in the significant increase in lifetime in glycerol to 0.5 ns.

**Figure 1:**
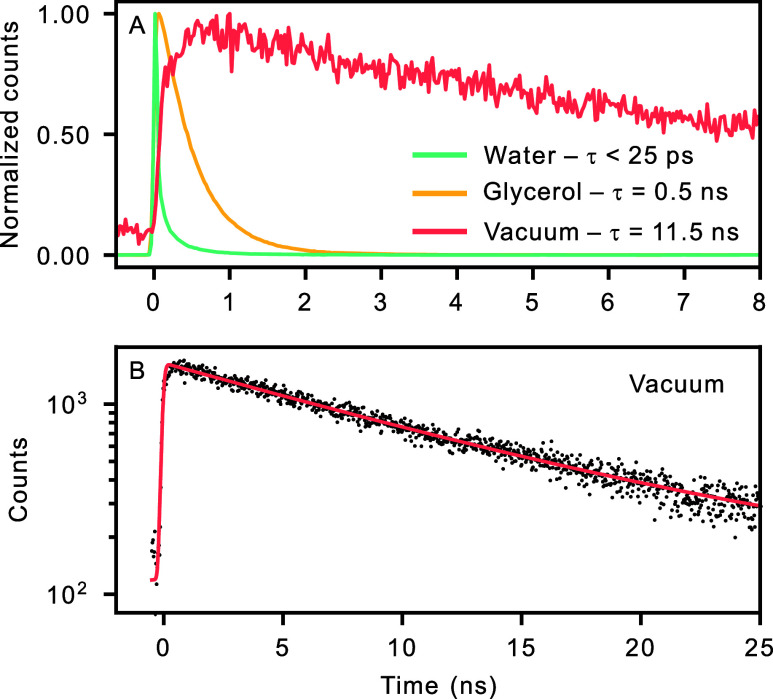
Fluorescence decay curves of TO in (A) water, glycerol, and in vacuum. (B) Fluorescence decay curve in vacuum. The excitation wavelength was 460 nm in gas-phase and 480 nm in solution-phase experiments. All traces are fitted with a (multi)­exponentially modified Gaussian function to extract the excited-state lifetimes. Complete details are found in the SI (Figures S7–S9).

In the gas phase, where there is no external friction, TO is expected to rotate freely, which would result in a short excited-state lifetime as in solution phase. However, gaseous TO displays surprisingly long-lived fluorescence (Figure 1B). At RT, the excited-state lifetime of TO is 11.5 ± 0.2 ns. Removing solvent altogether thus leads to a > 500- or 25-fold increase in lifetime compared to TO in water or glycerol, respectively. This clearly indicates a very stable fluorescent excited-state geometry, and that the ultrafast internal conversion (IC) pathway known from solution phase is not inherently open. At 100 K, we similarly measure a lifetime of 11 ± 1 ns (Figure S6). Single-exponential decay and identical lifetimes at 100 K and at RT imply a barrier to deactivation at least on the order of magnitude of the ion’s internal energy at RT. The internal energy of TO is calculated to be 0.43 and 0.05 eV at 300 and 100 K (using ground-state frequencies at PBE0-D4/def2-TZVPD level of theory, *vide infra*).

Higher laser fluence leads to significant fragmentation of TO (Figures S20 and S21). We ascribe this to ground-state heating and potential photoisomerization into a nonfluorescent conformer, which fragments after photoexcitation (see SI). We noted no apparent dependence of the lifetime on the power (5 to 15 mW) and excitation energy (445 to 475 nm) within experimental uncertainty. Previous studies have shown that triplet states can influence fluorescence measurements of trapped gas-phase ions.^9^ Dark triplet states of rhodamine cations were quenched with oxygen collisions to enhance fluorescence.^10^ The triplet state of TO was previously described as a photoinduced generator of singlet dioxygen.^11^ In our experiment, the detected emission is assigned to fluorescence (S_1_) and not phosphorescence from a triplet state, as shown by the limited Stokes shift.

The loss of fluorescence in solution was previously rationalized by SA-CASSCF calculations on the much-reduced symmetrical model system (H_2_N-(CH)_3_-NH_2_^+^).^12,13^ Such calculations show barrierless twisting on the excited-state (S_1_) PES from the locally excited (LE) state toward a twisted intramolecular charge transfer (TICT) state. A conical intersection close to the TICT minimum connects the S_1_ and S_0_ PESs and facilitates rapid internal conversion to the latter. While reproducing solution-phase results, this model system does not correctly describe our gas-phase findings; it seems inadequate for the description of asymmetric cyanine dyes, such as TO. A barrier to deactivation in TO was previously proposed from semiempirical calculations,^14,15^ but was not reproduced with TD-DFT.^16^ In either case, the oscillator strength decreases as the system twists, and fluorescence is therefore expected to occur only when ψ < 60°.^14^

To support the existence of an excited-state barrier to deactivation, we measured the absorption (fluorescence excitation) and dispersed fluorescence spectra of the bare TO ion at 100 K at LUNA2 (Figure 2A and Figure S2). The absorption spectrum shows a maximum at 475 ± 1 nm and clear vibronic features at 465 and 447 nm, while the fluorescence spectrum exhibits a maximum at 490 ± 2 nm with a vibronic shoulder at 521 nm. The spectral mirror symmetry supports similar ground- and excited-state geometries (Figures S3 and S4). Furthermore, spectra at RT show similar band maxima also indicating a stable excited-state local minimum structure (Figure S5). The Stokes shift for the bare TO ion is around 600 cm^–1^. For comparison, at low temperature, rigid systems like oxazines display Stokes shifts of only 14–50 cm^–1^,^17^ an order of magnitude less than TO. In systems with barrierless excited-state twisting in the gas phase, like thioflavin T and thioflavin X, the shift is significantly larger (over 3000 cm^–1^), and emission spectra are broad and unresolved.^18^ Hence, TO’s 600 cm^–1^ Stokes shift suggests some excited-state relaxation, without relaxing to the ground state through a twisted geometry.

**Figure 2:**
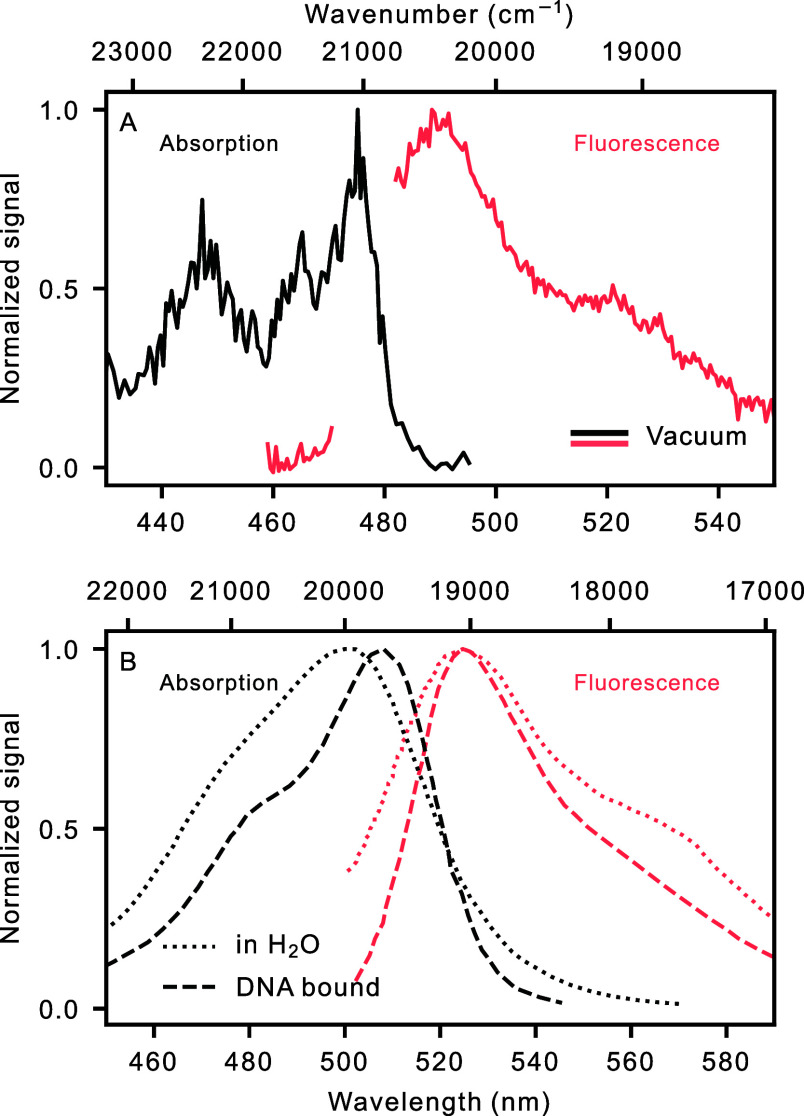
Absorption and fluorescence spectra of isolated TO ions at 100 K in the gas phase (A) and of the TO monomer in water and bound to DNA (B). Solution-phase spectra were digitized from published references.^1,16^ The gas-phase dispersed fluorescence spectrum was recorded without laser filters at an excitation wavelength of 476 nm by delaying the spectrometer’s iCCD camera such that the 5 ns laser pulse has passed.

Previously published spectra of the TO monomer in water^1,16^ and bound to DNA^1^ are shown in Figure 2B for comparison with gas-phase measurements. The absorption maximum for the monomer is 501 nm and redshifts by 300 cm^–1^ to 508 nm upon binding to DNA. In either case, the fluorescence spectra show maxima around 525 nm. The main effect of binding is a narrowing of the spectra, suggesting a more localized minimum near the Franck–Condon region. Compared to the gas phase, DNA-bound TO absorption and fluorescence spectra are red-shifted by around 1400 cm^–1^. Such solvent shifts are expected for π → π* transitions.^19^ Apart from the shift, the spectral shapes are similar to those measured in the gas phase, and the Stokes shift is also around 600 cm^–1^ upon binding to DNA. Altogether, this implies that the gas-phase emissive geometry of TO is similar to that of DNA-bound TO, further supporting an intrinsic barrier to twisting in S_1_ for gaseous TO.

To support the experimental findings, we performed quantum-chemical calculations of the ground and excited states of TO. Calculations were performed with ORCA 6.1,^20^ and the conformational space was sampled using the GOAT algorithm^21^ in ORCA with the GFN2-xTB method.^22^ Throughout, (TD-)­DFT calculations were conducted using the PBE0-D4 functional and the def2-TZVPD basis set, unless stated otherwise. PBE0 was previously used for cyanine dyes.^23^ Ground-state calculations (Figure S10 and Tables S1 and S2) revealed slight twisting of the lowest-energy conformer, here denoted *trans* (ϕ = −176°, ψ = 8°), with the *cis* conformer (ϕ = −179°, ψ = −147°) 0.17 eV above in energy (zero-point corrected). The very soft nature of the ψ-torsional motion (a few tens of cm^–1^ torsional mode) makes the ground-state geometry sensitive to the method used. This foreshadows challenges in accurately capturing the excited-state behavior as the molecule undergoes torsional motion. Indeed, as found previously,^16^ TD-DFT is unable to capture a fluorescent minimum on the excited-state PES, contrasting our current gas-phase experiments (Figure S12). Instead, TD-DFT provides an optimized excited-state geometry in which both π-systems are perpendicular to each other and negligible oscillator strength for the S_1_–S_0_ transition (Figure S11).^16,24^

Due to the lack of a local fluorescent minimum on the excited-state PES at TD-DFT level, absorption and fluorescence Franck–Condon (including Herzberg–Teller) spectra were simulated with the AHAS (Adiabatic Hessian After a Step) model implemented in ORCA, which from a ground-state geometry and Hessian can estimate the excited-state geometry and Hessian via a step on the excited-state PES.^25^ Computed spectra at 100 K are seen in Figure 3. More details including a comparison between different functionals, basis sets, and temperatures are found in the SI (Figures S13–S17). These align well with the experimental results, both in reproduction of general shapes but also in absolute transition energies. As the AHAS method assumes similar S_0_ and S_1_ geometries, the matching spectra suggest a PES as depicted in Figure 3, where fluorescence occurs from a local minimum on the excited-state PES. The local S_1_ minimum is further corroborated by SA2-CASSCF­(2,3)/def2-svp calculations with NEVPT2 dynamic correlation ((2,3) is the minimum active space to describe the twist motion,^26^ see SI including Figures S18 and S19) on excited-state TD-DFT relaxed-scan geometries along the ψ dihedral angle. These calculations furthermore predict *cis* conformers to be nonfluorescent. Therefore, in the development of novel dyes, the exploration of spectral properties of TO derivatives may be performed (using the AHAS model) even if TD-DFT fails to locate the stable excited-state locally excited geometry.

**Figure 3:**
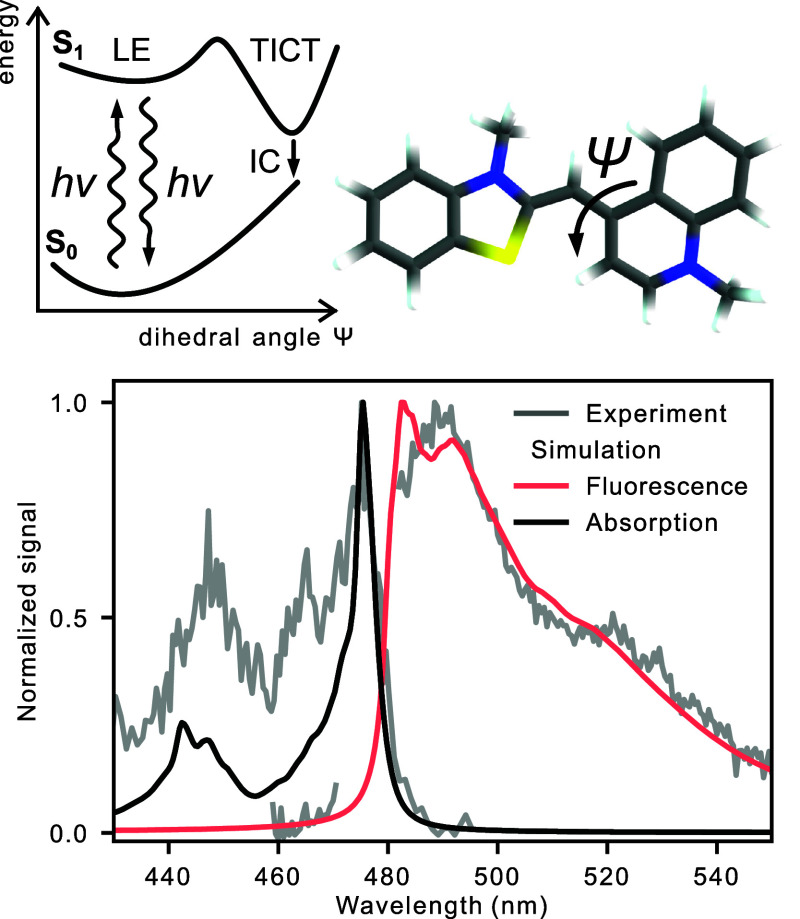
(Top) Schematic of the PES along the ψ dihedral angle. (Bottom) The simulated absorption and fluorescence spectra plotted together with the experimental spectra. The calculated absorption and fluorescence spectra are red-shifted by 2 nm (100 cm^–1^) and 7 nm (300 cm^–1^), respectively, to match the experimental spectra.

Comparatively, the green fluorescent protein (GFP) chromophore anion shares a similar twist motion and deactivates quickly through a conical intersection if rotation is unhindered in S_1_. If removed from the protein, the chromophore anion only exhibits fluorescence in vacuum below 150 K.^27^ Calculations have also proven challenging with TD-DFT functionals yielding inconsistent results.^27^ Even at CASSCF level,^28^ the twist barrier is hard to calculate requiring dynamic correlation at high level.^29^

Our experimental and computational findings for TO suggest an intrinsic excited-state barrier to rotation toward the TICT state. Why is this not observed in solution? Notably, polar solvents can directly influence the barrier to rotation.^30^ The locally excited state is stabilized by delocalization of orbitals which favors a planar structure, while solvent molecules increase charge localization favoring one resonance form over the other (Scheme 1), destabilizing the locally excited state and further stabilizing the TICT state. Solution-phase studies of poly methine cyanines have found excited-state rotational barriers to be solvent and bridge-length dependent.^14^ McConnell et al.^31^ argued that a barrier can generally exist for monomethine cyanines in sufficiently nonpolar solvents. The gas phase is therefore the ideal environment for studying intrinsic excited-state barriers of dyes, as such an experiment is challenging to perform in solution due to the impact of counterions and limited solubility of ionic dyes in nonpolar solvents.

In summary, our results reveal a surprising interplay between TO and its environment: fluorescence is intrinsic to the ion, but quenched in solution, and reestablished in a rigid environment. As such, it is not a rigid environment that turns on fluorescence, but water, and maybe solvation with polar solvents in general, that turns it off. Understanding the effect of hydration requires more work but is likely related to water stabilizing the TICT state, which would reduce or even remove the barrier along the torsional coordinate, leading to near-barrierless deactivation in solution. The results presented here serve as benchmarks for future higher-level calculations and modeling to fully understand the intriguing photophysics of TO. Meanwhile our current (TD-)­DFT workflow can reproduce spectral properties for future dye development.

## Supplementary Material



## Data Availability

The data that support the findings of this study are openly available in ETH Zurich Research Collection at 10.3929/ethz-c-000801361.

## Notes

Detailed descriptions of the experimental and computational procedures, mass spectra, experimental and computed absorption and fluorescence spectra, experimental fluorescence lifetime decay curves, and xyz-coordinates (PDF)
