# Supplementary material for: Thiazole Orange Fluoresces Freely: No Rigid Environment Required
Source: J Phys Chem Lett. 2026 Jul 18;17(30):8643–7. doi: 10.1021/acs.jpclett.6c02059 (PMC13430677; doi:10.1021/acs.jpclett.6c02059)
Supplement: Supplementary file 1 [file jz6c02059_si_001.pdf]

Supplementary Information

Thiazole Orange Fluoresces Freely: No Rigid Environment Required

Kim Greis,‖ [a] Thomas Toft Lindkvist,‖ [b] Michael Schreivogel,[a] Iden Djavani-Tabrizi,[b] Franco Molina,[b] Steen Brøndsted Nielsen,*[b] Renato Zenobi*[a]

(a) ETH Zürich, Laboratory of Organic Chemistry, Department of Chemistry and Applied Biosciences, CH-8093 Zürich, Switzerland
(b) Aarhus University, Department of Physics and Astronomy, DK-8000 Aarhus, Denmark

‖ These authors contributed equally.

* Correspondence to: sbn@phys.au.dk
zenobi@org.chem.ethz.ch

Table of Contents 2

Experimental details 3
ETH Zürich setup 3
LUNA2 setup 3
Mass Spectrometry 4
Excitation and fluorescence emission spectra 5
Fluorescence lifetime measurements 7
Computational Results 9
Computed geometries 9
(TD)-DFT potential energy surfaces 10
Calculated Franck-Condon (Herzberg-Teller) spectra 11
CASSCF calculations 14
Photofragmentation 16
Computed xyz-coordinates 18

Experimental details

ETH Zürich setup

The gas-phase experiments at ETH Zürich were performed using a modified LCQ ion trap mass spectrometer (Thermo Fisher Scientific, Waltham, MA, USA). Ions were generated via nanoelectrospray ionization. An aqueous solution of TO (100 μM) was loaded into a glass capillary (1.5 μm inner diameter) made using a micropipette puller (P-1000, Sutter Instrument). The spray voltage of 1.0–1.2 kV was provided via a platinum wire electrode inserted into the capillary. The generated positively charged ions were directed into the 3D ion trap of hyperbolic geometry via octupole ion guides. The mass spectrometer is operated in tandem MS (MS/MS) mode to isolate the ion of interest. In each duty cycle ions are accumulated for 1 s followed by irradiation for 1 s.

The trap was modified to allow for laser excitation and fluorescence collection (2.3% solid angle), as previously described in detail (P Tiwari *et al. J. Am. Soc. Mass. Spectrom.* **2021**, *32*, 187-197). Two opposing 1.5-mm apertures in the ring electrode provide optical access for the excitation laser. A 5.0 mm lens mounted into the ring electrode allows fluorescence to be collected perpendicular to the laser path, minimizing background interference and directing the light toward the detection system.

The excitation light was generated from an 80-MHz Ti:sapphire laser (Mai Tai, Spectra-Physics, USA), which was frequency-doubled via a second harmonic generator (UHG, Spectra-Physics, USA). These pulses were redirected via optical mirrors to a pulse picker (350-105-UV, Conoptics), to reduce the repetition rate to 26.7 MHz, ensuring sufficient excited-state relaxation between consecutive excitations events.

In the TCSPC experiments (gas phase and solution phase alike), laser-induced fluorescence is collected by a single-photon avalanche diode (PicoQuant GmbH, Berlin, Germany) and time-tagged using a PicoQuant TimeHarp 260. The lifetime data was recorded using Time Harp software (v3.0.0.0, PicoQuant GmbH) and later analyzed in a python routine.

LUNA2 setup

Cold-ion fluorescence spectroscopy was performed at Aarhus University with the home built LUNA2 instrument (C Kjær, *et al. Rev. Sci. Instrum.* **2021**, *92*, 033105). Ions were generated in electrospray ionization, bunched in an octupole pretrap before being sent at 20 Hz (synchronized with the laser excitation) to the fluorescence cell. Here, the ions were trapped in a custom cylindrical ion trap, with one "transparent" mesh-grid end-cap to allow a collection solid angle of around 10%. The trapped ions were excited using light from a tunable ns laser (EKSPLA, 20 Hz, 3-5 ns pulse width, <5 cm^{-1} linewidth). Trapped ions were furthermore mass selected after each irradiation event to remove impurities and photofrag-

ments. The emitted fluorescence was directed into a photomultiplier tube (Thorlabs PMT1001/M) to measure the total fluorescence yield in a fluorescence excitation measurement, or into a spectrometer combined with an intensified CCD (iCCD) camera (Andor iStar 320) to resolve the dispersed fluorescence spectrum. The intensifier in the iCCD camera was synchronized with the laser so as to only intensify for 100 ns around the excitation. When no filters were used to remove scattered laser light, the intensifier was instead delayed to hereby reduce the contribution of scattered laser light.

Mass Spectrometry

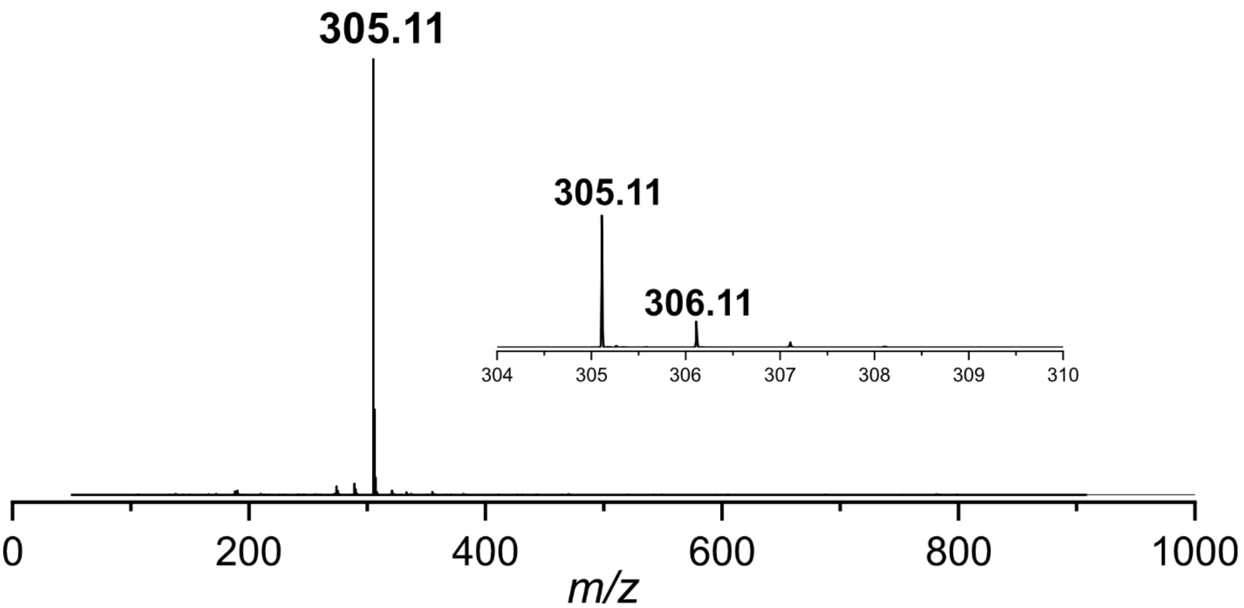

Figure S1. Full nESI mass spectrum of thiazole orange (100 µM, H_2O). The isotope pattern in the inset confirms the formation of thiazole orange cation monomers and that dimers are not formed in a measurable quantity.

Excitation and fluorescence emission spectra

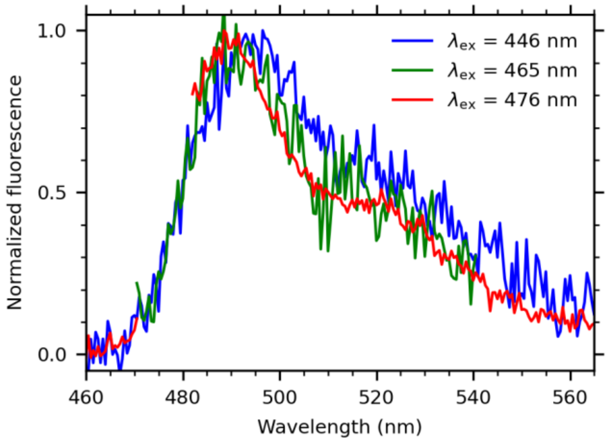

Figure S2. Dispersed fluorescence spectra at different excitation wavelengths measured at 100 K with LUNA2. Higher excitation energy (shorter wavelengths) lead to broadening and redshift corresponding to an increase in internal energy (heating) of the excited state.

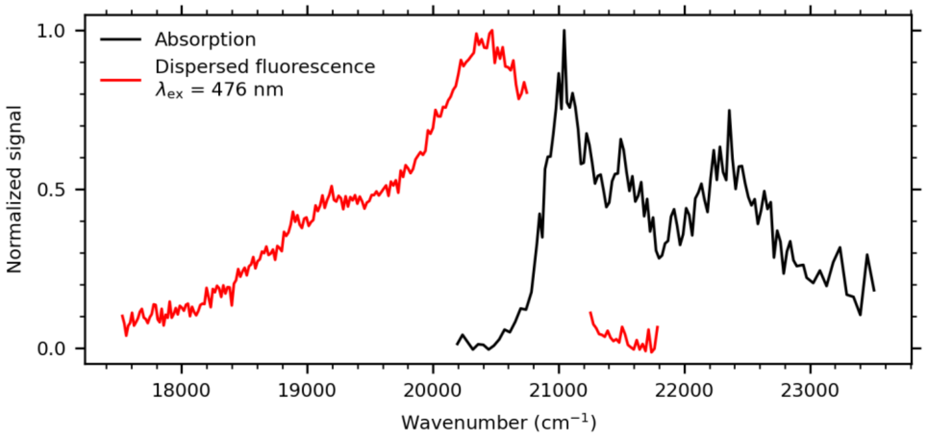

Figure S3. Fluorescence excitation and dispersed fluorescence spectra at 100 K as a function of energy. The two spectra show mirror symmetry as also seen when the two spectra are plotted as a function of the absolute shift from their respective maxima (seen in the figure below). The dispersed fluorescence spectrum is broader than the fluorescence excitation spectrum as the latter is limited by the laser band-width while the former is broadened due to the large slit-width necessary in these gas-phase fluorescence measurements.

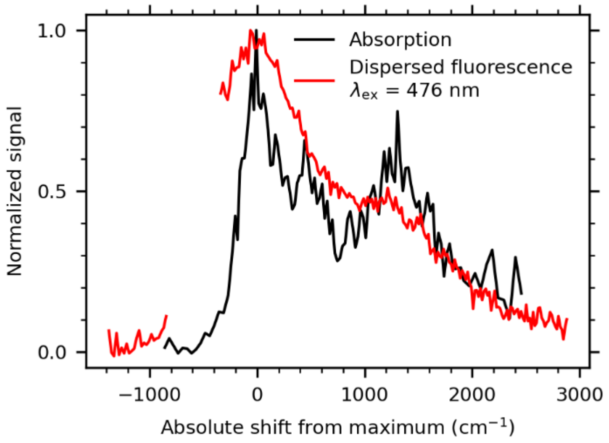

Figure S4. Fluorescence excitation and dispersed fluorescence spectra at 100 K as a function of the absolute shift from their respective maxima. The ground- and excited-state geometries must be similar due to the common vibronic features in the spectra (mirror symmetry).

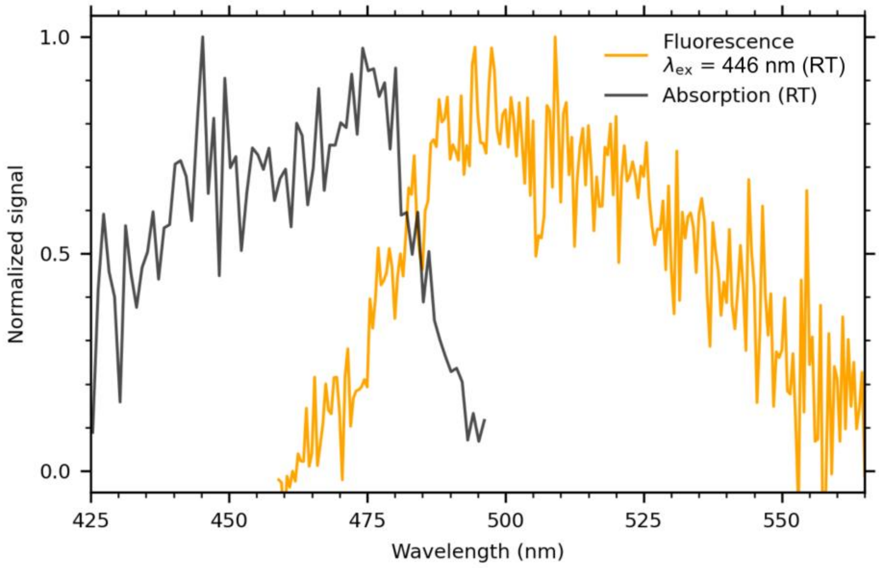

Figure S5. Fluorescence excitation (absorption) and dispersed fluorescence spectra at 298 K (RT).

Fluorescence lifetime measurements

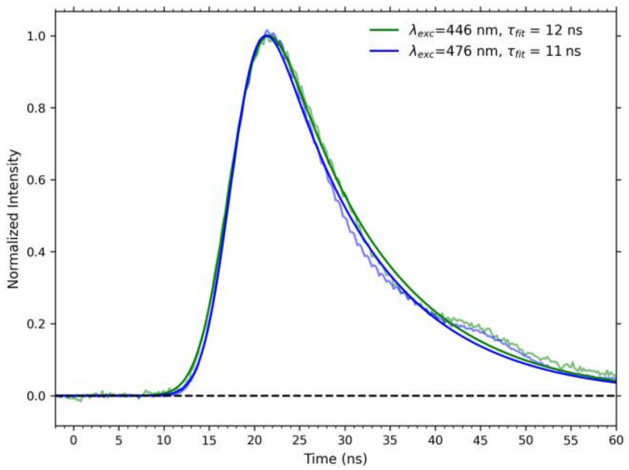

Figure S6. Temporal trace of the fluorescence signal as recorded on the PMT in the LUNA2 experiment at 100 K. An excited-state lifetime is extracted via a fit to an exponentially-modified Gaussian. The Gaussian component includes the pulse width of the laser pulse as well as the bandwidths of the PMT amplifier and the oscilloscope. The uncertainty of the measurement is around 1 ns and there is therefore no significant difference between the two excitation wavelengths.

(Multi-) exponentially-modified Gaussian fits to time-resolved fluorescence traces recorded in TCSPC experiments are seen in Figure S7-S9 (ETH Zürich setup).

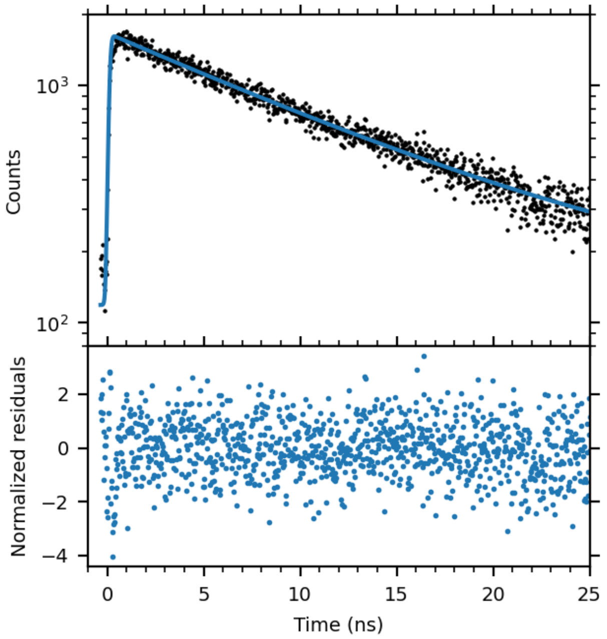

Figure S7. Time-resolved fluorescence of room-temperature TO in the gas phase recorded in a TCSPC experiment. In the gas phase TO shows a lifetime of 11.5 ± 0.2 ns.

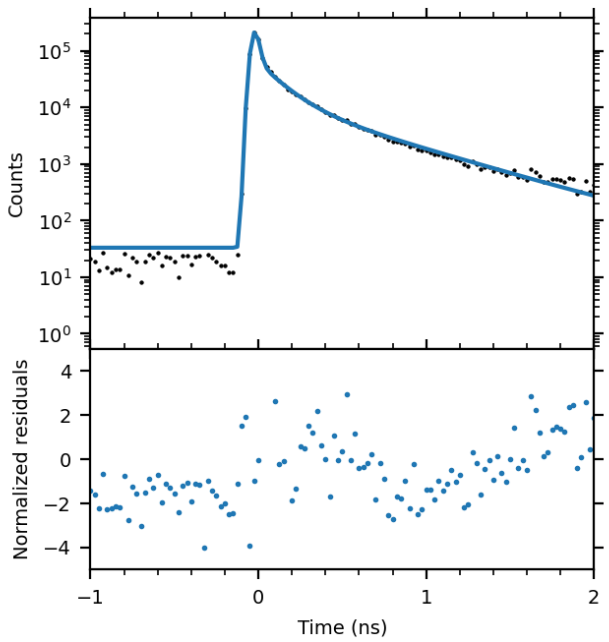

Figure S8. Time-resolved fluorescence of TO in water. Three lifetime components (<25 ps, 130 ps and 0.5 ns) were needed to adequately fit the trace.

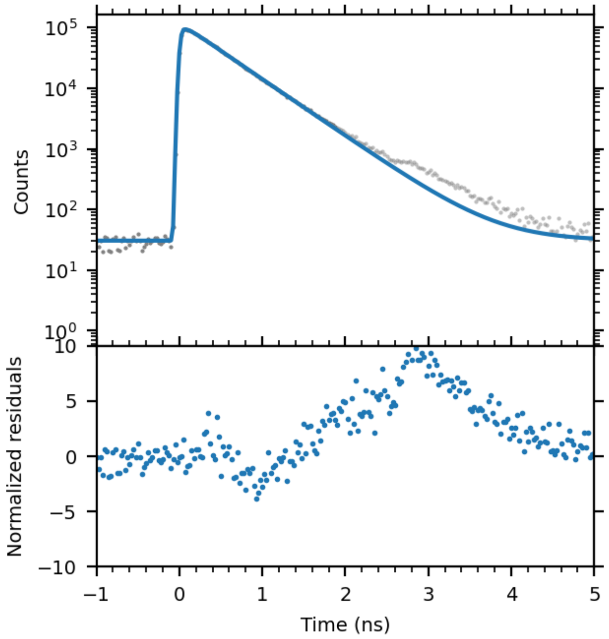

Figure S9. Time-resolved fluorescence of TO in glycerol shows a lifetime of 0.5 ± 0.1 ns. The given uncertainty is a conservative estimate as the fit was truncated to ~2 ns due to the spurious increase in signal observed thereafter.

Computational Results

Computed geometries

Four canonical conformations are possible with the two central dihedral angles (ϕ, ψ). In our nomenclature, the ϕ dihedral angle yields E/Z forms and the ψ dihedral angle gives rise to *trans*/*cis* forms. In the main text only the *trans*/*cis* of the Z forms are considered as *trans*-Z is the only thermally relevant form and as we only consider the ψ dihedral angle relevant in the excited state deactivation.

See Table S1 for the relative energies, dihedral angles and vertical excitation energies of the four forms. The geometries are given further below. Table S2 shows the dihedral angles of *trans*-Z computed with different DFT functionals and basis sets.

Table S1. Ground-state energies, vertical excitation energies (VEE), and oscillator strengths of the four ground-state conformers. Calculated with PBE0-D4/def2-TZVPD. Only the lowest-energy *trans*-Z conformer is relevant in a thermal equilibrium.

	Zero-point corrected relative energies	Dihedral angles	VEE $S_0 \rightarrow S_1$	Oscillator strength
trans-Z	0 eV	$\phi = -176°, \psi = 8°$	3.13 eV (396 nm)	1.20
trans-E	0.167 eV	$\phi = -37°, \psi = -15°$	3.12 eV (397 nm)	1.20
cis-Z	0.169 eV	$\phi = -179°, \psi = -147°$	2.89 eV (429 nm)	0.75
cis-E	0.244 eV	$\phi = 32°, \psi = -158°$	3.02 eV (411 nm)	0.93

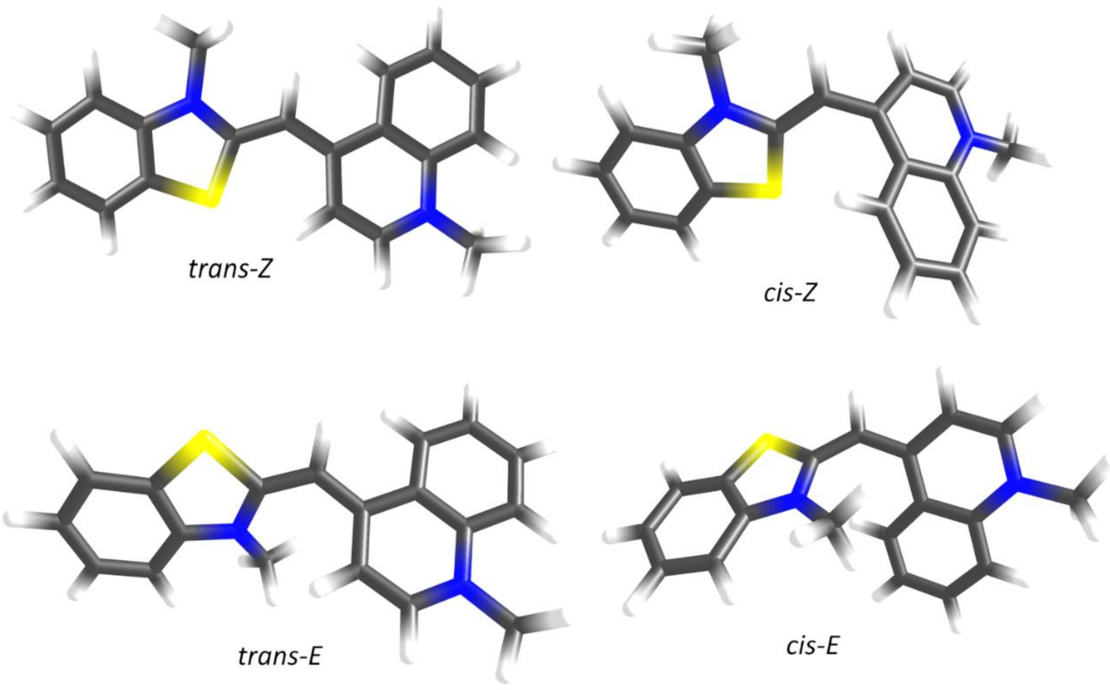

Figure S10. Four ground-state conformations calculated with PBE0-D4/def2-TZVPD. The xyz-coordinates of these geometries can be found at the end of this document.

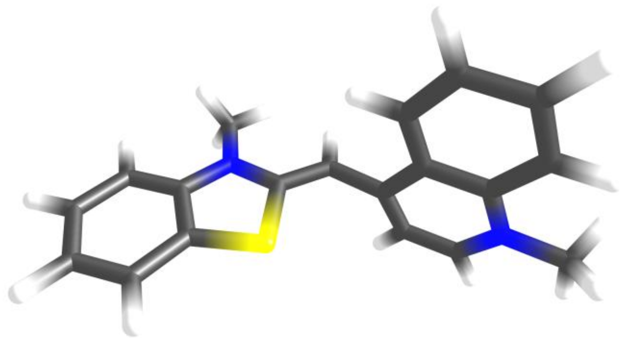

Figure S11. S_1 twisted-Z minimum structure ($\boldsymbol{\phi} = \mathbf{180}°$, $\boldsymbol{\psi} = \mathbf{96}°$) calculated with PBE0-D4/def2-TZVPD.

Table S2. Computed dihedral angles $\boldsymbol{\phi}$ and $\boldsymbol{\psi}$ for lowest-energy ground-state TO conformer (trans-Z, see Figure S10). Results indicate that the exact dihedral angles are method dependent.

Method	ϕ	ψ
PBE0-D4/def2-TZVPD	-176°	8°
PBE0-D4/def2-SVPD	-176°	7°
ωB97X /def2-TZVPD	-176°	10°
ωB97X/def2-SVPD	-180°	0°
ωB97XD/aug-cc-pvdz	-180°	0°
r^{2}SCAN-D4/def2-TZVPD	-176°	9°
r^{2}SCAN-D4/def2-SVPD	-176°	8°
MP2/def2-TZVPD	-176°	10°

(TD)-DFT potential energy surfaces

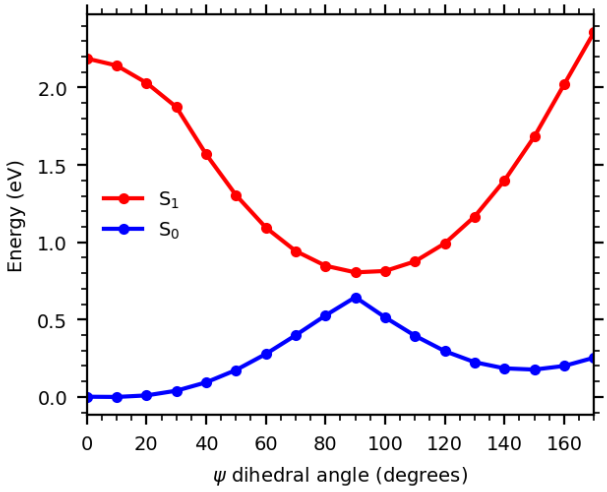

Figure S12. Computed S_0 and S_1 potential energy surfaces along the $\boldsymbol{\psi}$ dihedral angle at (TD-)DFT level with PBE0-D4/def2-TZVPD connecting the trans-Z and cis-Z conformers.

Calculated Franck-Condon (Herzberg-Teller) spectra

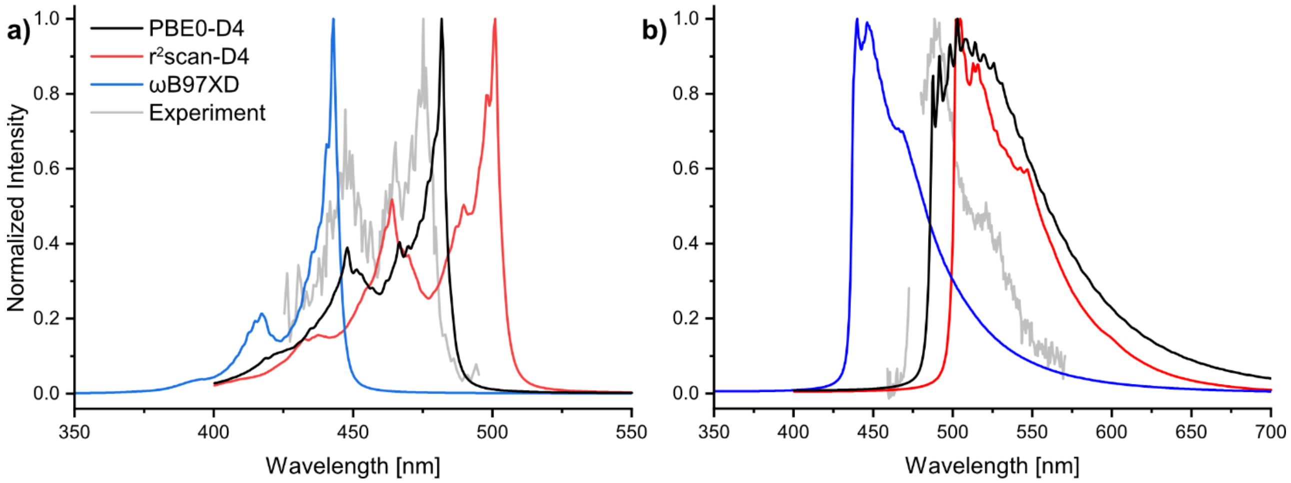

Figure S13. Computed (a) UV-vis absorption and (b) fluorescence spectra of the thiazole orange cation. The spectra were computed at the PBE0-D4/def2-SVPD (black), r^2scan-D4/def2-SVPD (red), and ωB97XD/def2-SVPD (blue) levels of theory using the AHAS method at 100 K. The experimental spectrum is shown in the gray trace. Spectra include Herzberg-Teller effects, do not include Duschinsky rotations, and frequencies below 70 cm^{-1} were not considered.

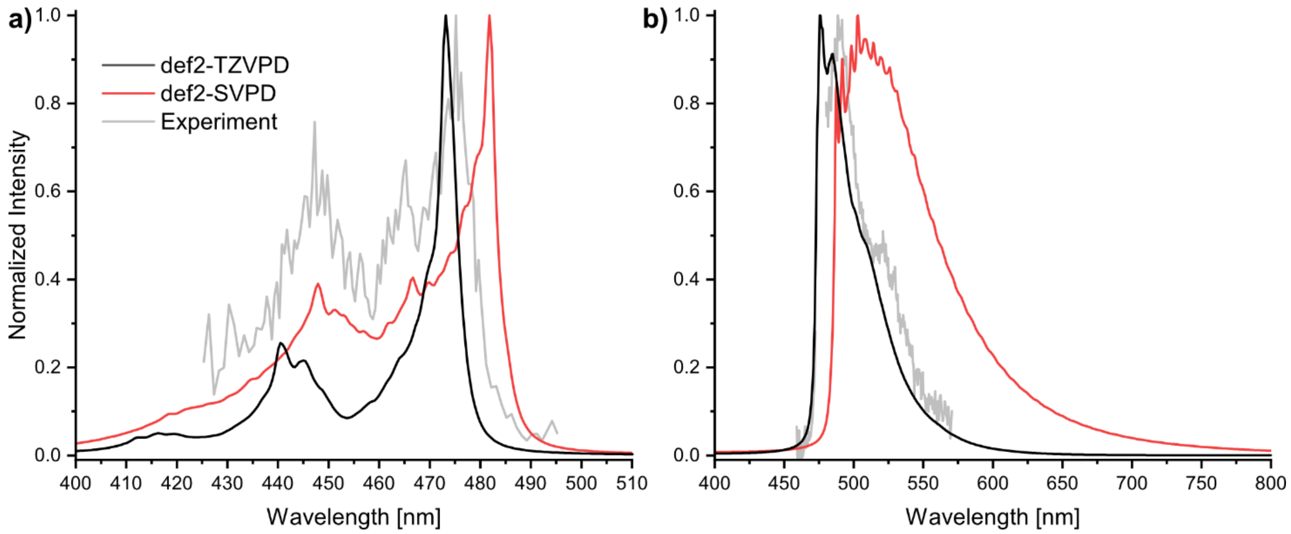

Figure S14. Computed (a) UV-vis absorption and (b) fluorescence spectra of the thiazole orange cation. The spectra were computed at the PBE0-D4/def2-TZVPD (black) and the PBE0-D4/def2-SVPD levels of theory using the AHAS method at 100 K. The experimental spectrum is shown in the gray trace. Spectra include Herzberg-Teller effects, do not include Duschinsky rotations, and frequencies below 70 cm^{-1} were not considered.

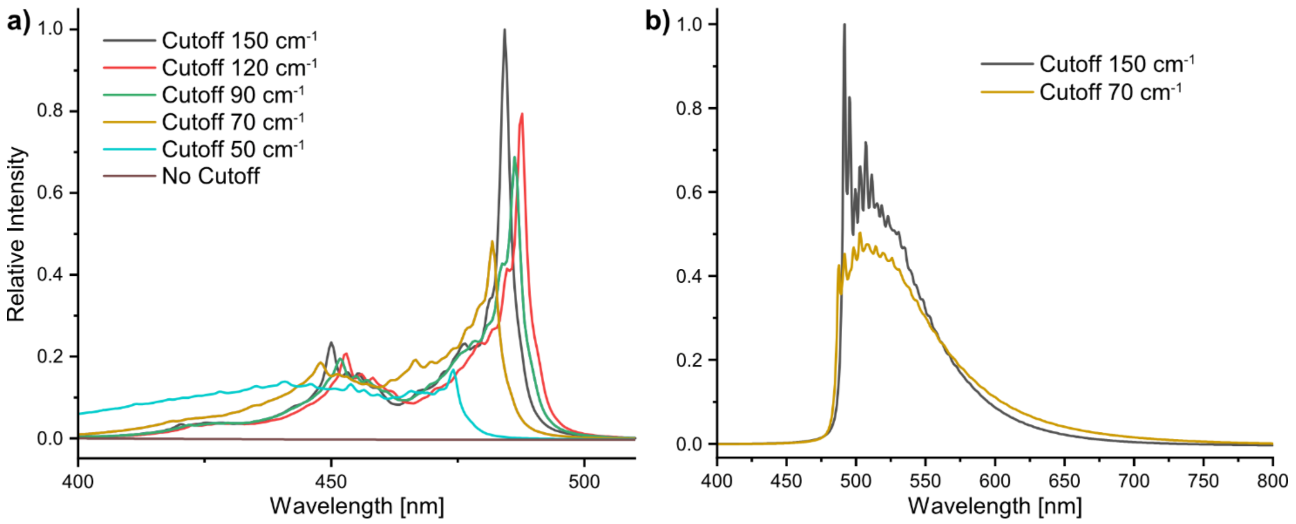

Figure S15. Computed (a) UV-vis absorption and (b) fluorescence spectra of the thiazole orange cation. The spectra were computed at the PBE0-D4/def2-SVPD level of theory using the AHAS method at 100 K, with frequencies below (black) 150 cm^{-1}, (red) 120 cm^{-1}, (green) 90 cm^{-1}, (yellow) 70 cm^{-1}, and (blue) 50 cm^{-1} cut off. The brown trace, in which all frequencies were considered, does not contain any spectral signals. Spectra include Herzberg-Teller effects and do not include Duschinsky rotations.

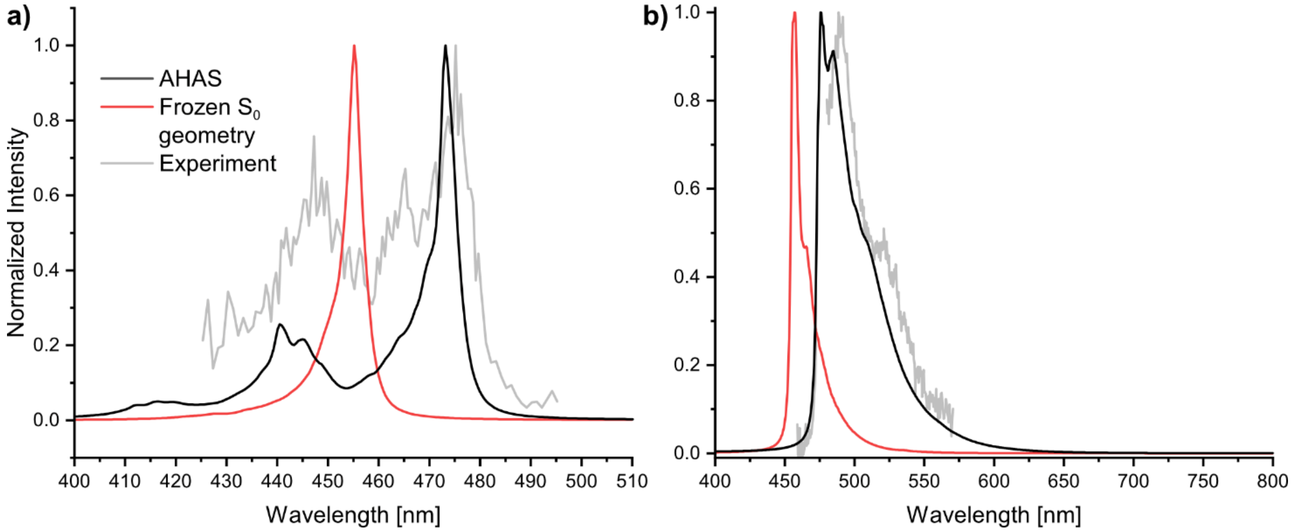

Figure S16. Computed (a) UV-vis absorption and (b) fluorescence spectra of the thiazole orange cation. The spectra were computed at the PBE0-D4/def2-TZVPD using the AHAS method (black) or assuming the frozen S_0 geometry as excited state geometry (red) at 100 K. The experimental spectrum is shown in the gray trace. Spectra include Herzberg-Teller effects, do not include Duschinsky rotations, and frequencies below 70 cm^{-1} were not considered.

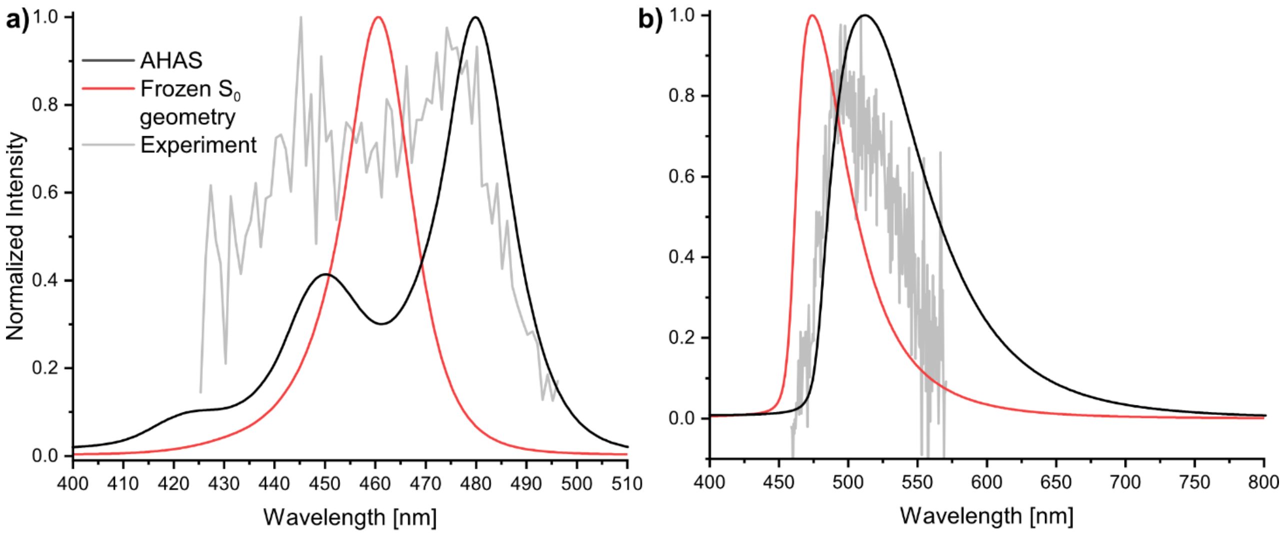

Figure S17. Computed (a) UV-vis absorption and (b) fluorescence spectra of the thiazole orange cation. The spectra were computed at the PBE0-D4/def2-TZVPD using the AHAS method (black) or assuming the frozen S_0 geometry as excited state geometry (red) at 298 K. The experimental spectrum is shown in the gray trace. Spectra include Herzberg-Teller effects, do not include Duschinsky rotations, and frequencies below 70 cm^{-1} were not considered.

CASSCF calculations

Calculations using SA2-CASSCF(2,3) with NEVPT2 were performed on TD-DFT geometries from a relaxed scan of the ψ dihedral on the excited-state PES (Figure S18). There is a clear local minimum close to the ground-state minimum. This agrees with the experimental findings.

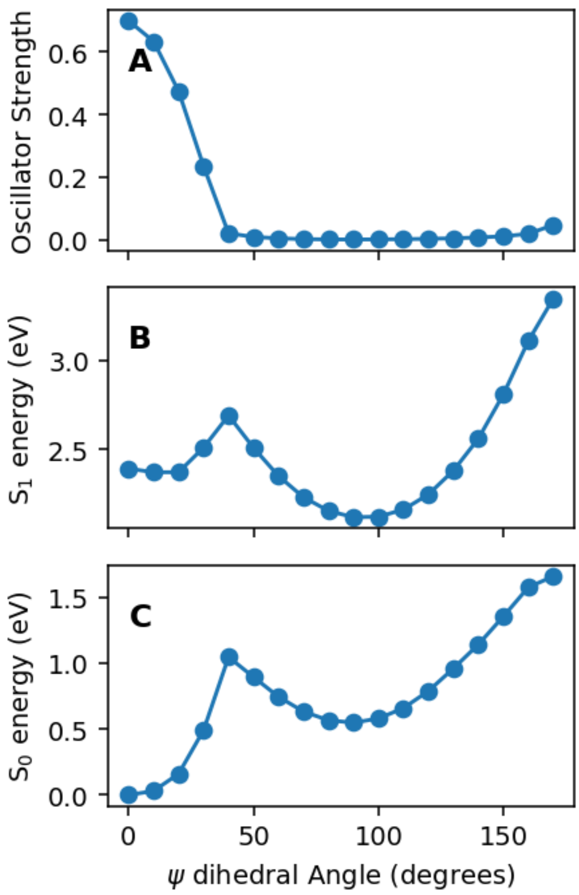

Figure S18. Excited- and ground-state properties as a function of the ψ dihedral angle computed using SA2-CASSCF(2,3) with NEVPT2 on TD-DFT geometries. (A) The oscillator strength of the S_1-S_0 transition. (B) S_1 energy. (C) S_0 energy.

As argued previously, (NH List, *et al. Chem. Sci.* **2022**, *13*, 373-385. & S Olsen, *et al. J. Chem. Phys.* **2009**, *131*, 234306) two electrons in three orbitals of binding, anti-binding and nonbinding character across the methine bridge is the minimum active space needed to describe this twist. Figure S19 shows the orbitals used.

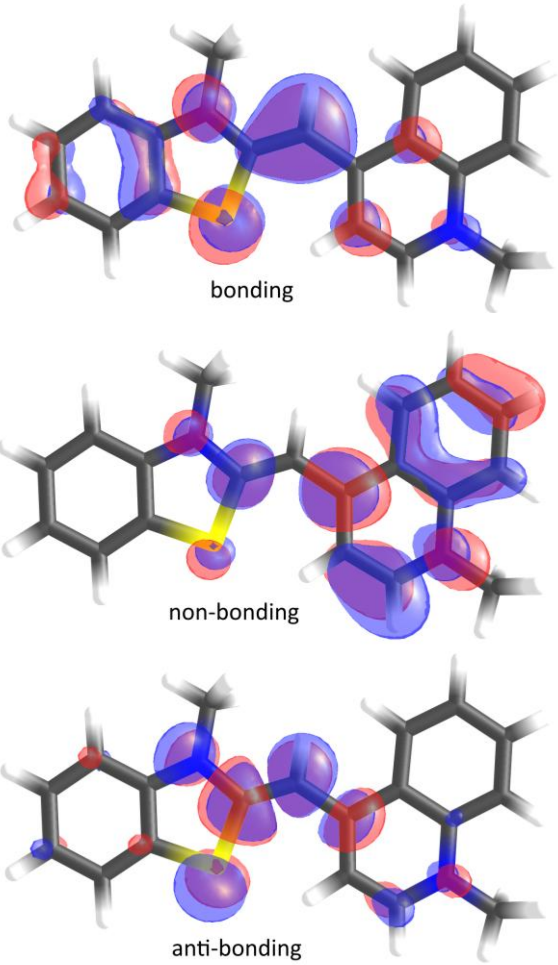

Figure S19. Active-space orbitals used in the NEVPT2/SA2-CASSCF(2,3) calculations given at $\boldsymbol{\psi} = \mathbf{0}°$.

Photofragmentation

Fragmentation mass spectrum and fragmentation as a function of laser power are seen in Figures S20 and S21, respectively. Clearly, the fluorescence quantum yield is not 100 %. So, while the lifetime in the excited state is long, longer than for other ions we have previously measured, there is a significant non-radiative decay component. Even with a very small chance of dissociation, in the ETH Zürich setup where ions are irradiated at 26.6 MHz, the many excitation events can quickly cause most of the population to dissociate.

Furthermore, the CASSCF calculated S_1 PES (Figure S18) only finds a local, fluorescent, minimum for the *trans-Z* conformer. The calculations therefore find the *cis-Z* conformers to be non-fluorescent. Thus, if the barrier to $\boldsymbol{\psi}$-twisting on the excited-state PES is overcome, fast rotation to a conical intersection with the ground state could lead to photoisomerization. Conversion from the *trans-Z* to the non-fluorescent *cis-Z* conformer would then lower the apparent brightness of TO in the gas phase.

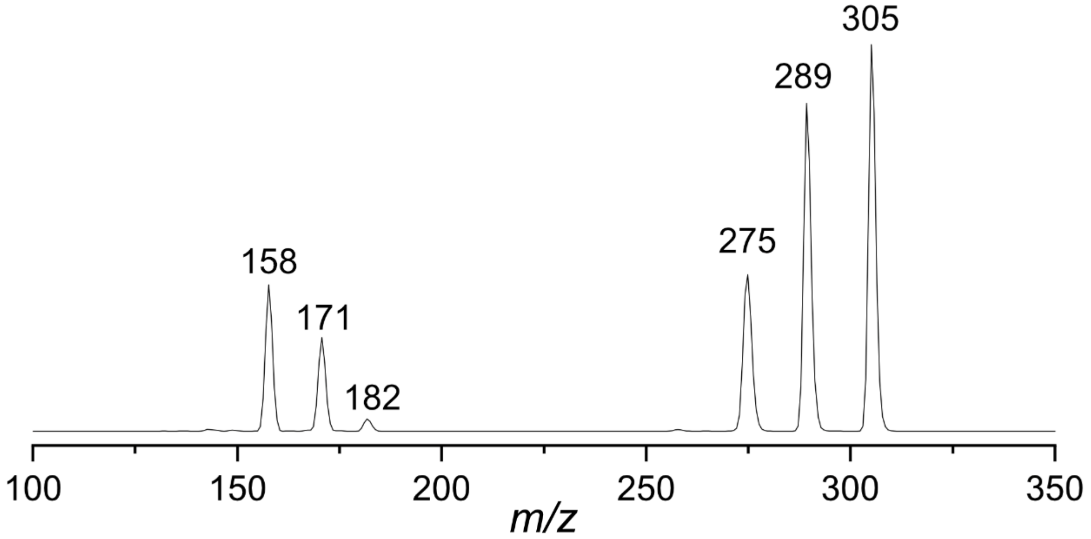

Figure S20. (+)-nESI visible photodissociation (460 nm) mass spectrum of thiazole orange (100 µM, H_2O).

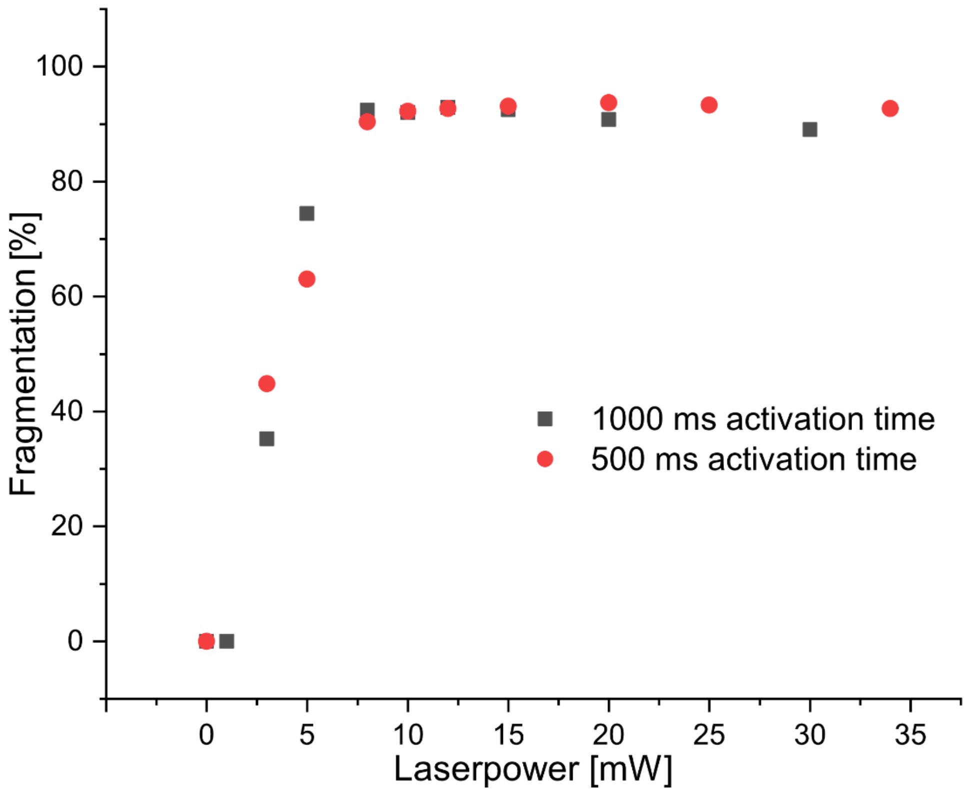

Figure S21. Fragmentation of TO as a function of laser power (460 nm). The amount of fragmentation is calculated by dividing the parent ion signal when the laser is switched on by the ion signal when the laser is switched off

Computed xyz-coordinates

Computed geometries of the four ground-state conformers with PBE0-D4/def2-TZVPD. First column has atomic numbers, second to fourth have xyz coordinates (in Ångstrøm).

Trans-Z

6	5.888747000	0.416514000	-0.041756000
6	5.811463000	-0.963296000	-0.212123000
6	4.751266000	1.191550000	0.100557000
1	6.858256000	0.898461000	-0.022178000
6	4.583385000	-1.599149000	-0.248043000
1	6.718388000	-1.544288000	-0.321256000
6	3.515753000	0.557332000	0.072412000
1	4.840147000	2.262851000	0.221733000
6	3.445709000	-0.822695000	-0.107586000
1	4.512856000	-2.670963000	-0.385037000
7	2.259726000	1.131841000	0.205602000
16	1.794678000	-1.345676000	-0.127906000
6	1.217302000	0.269663000	0.101517000
6	2.064210000	2.545555000	0.440640000
1	1.370042000	2.691939000	1.268731000
1	1.679223000	3.041794000	-0.453442000
1	3.013808000	2.996537000	0.710969000
6	-0.104724000	0.696630000	0.129717000
6	-1.279168000	-0.063914000	0.114456000
1	-0.216284000	1.768610000	0.144080000
6	-2.574918000	0.579252000	-0.013058000
6	-1.311922000	-1.466920000	0.235380000
6	-3.759800000	-0.195279000	-0.009779000
6	-2.487619000	-2.156217000	0.233262000
1	-0.415612000	-2.048905000	0.379449000
7	-3.683348000	-1.568510000	0.112420000
1	-2.500741000	-3.232785000	0.341577000
6	-4.888241000	-2.381998000	0.114122000
1	-5.432124000	-2.262012000	-0.823904000
1	-5.536087000	-2.105243000	0.947069000
1	-4.602588000	-3.425050000	0.223126000
6	-2.718523000	1.969031000	-0.158583000
6	-5.011685000	0.421086000	-0.132726000
6	-3.947531000	2.565816000	-0.278488000
1	-1.840581000	2.598141000	-0.188527000
6	-5.103097000	1.784321000	-0.261758000
1	-5.914348000	-0.172497000	-0.126991000
1	-4.019274000	3.640288000	-0.390783000
1	-6.076943000	2.248894000	-0.355654000

Trans-E

6	-5.428027000	1.251675000	0.517095000
6	-5.906488000	0.153371000	-0.193739000
6	-4.078093000	1.400767000	0.784145000
1	-6.122557000	2.005383000	0.866495000
6	-5.042800000	-0.823774000	-0.657441000
1	-6.967542000	0.062103000	-0.389145000
6	-3.210885000	0.422273000	0.320441000
1	-3.715257000	2.261314000	1.331447000
6	-3.690776000	-0.673642000	-0.395116000
1	-5.412478000	-1.678017000	-1.210526000
7	-1.833464000	0.372305000	0.510899000
16	-2.378555000	-1.703651000	-0.860863000
6	-1.226875000	-0.681085000	-0.077858000
6	-1.197106000	1.204128000	1.511187000
1	-0.276965000	0.723023000	1.834161000
1	-1.871742000	1.299670000	2.361976000
1	-0.964211000	2.198317000	1.125271000
6	0.129684000	-1.011424000	-0.077345000
6	1.217822000	-0.135508000	-0.130877000
1	0.328708000	-2.068648000	-0.192926000
6	2.570087000	-0.603372000	0.093369000
6	1.094165000	1.220468000	-0.503617000
6	3.667647000	0.266653000	-0.101047000
6	2.190928000	2.007846000	-0.678219000
1	0.131079000	1.639531000	-0.755339000
7	3.446102000	1.573100000	-0.491233000
1	2.094630000	3.033202000	-1.010659000
6	4.561547000	2.478543000	-0.708904000
1	4.171044000	3.443931000	-1.020825000
1	5.218856000	2.096013000	-1.491273000
1	5.133724000	2.611331000	0.210652000
6	2.840203000	-1.908320000	0.533191000
6	4.974093000	-0.192167000	0.107536000
6	4.123126000	-2.349852000	0.738117000
1	2.015207000	-2.577644000	0.736428000
6	5.195654000	-1.485715000	0.514150000
1	5.816834000	0.465968000	-0.048227000
1	4.301562000	-3.360929000	1.081740000
1	6.211287000	-1.827620000	0.671775000

Cis-Z

6	-5.556345000	-0.293899000	-0.324363000
6	-5.091176000	-1.491719000	-0.859656000
6	-4.682764000	0.675025000	0.138974000
1	-6.622219000	-0.110820000	-0.270429000
6	-3.732922000	-1.744560000	-0.948552000
1	-5.796553000	-2.232012000	-1.215360000
6	-3.320457000	0.421397000	0.058110000
1	-5.064188000	1.604473000	0.540658000
6	-2.858915000	-0.773302000	-0.491386000
1	-3.364454000	-2.671855000	-1.369191000
7	-2.280829000	1.238558000	0.478576000
16	-1.124971000	-0.815044000	-0.489599000
6	-1.037537000	0.761055000	0.229551000
6	-2.503582000	2.513167000	1.123578000
1	-3.484424000	2.506645000	1.592254000
1	-2.457222000	3.335389000	0.404726000
1	-1.754090000	2.664682000	1.899282000
6	0.104794000	1.535944000	0.399403000
6	1.453490000	1.198123000	0.208770000
1	-0.085911000	2.599467000	0.484955000
6	2.049976000	-0.095098000	0.436962000
6	2.337673000	2.199347000	-0.237330000
6	3.348338000	-0.372192000	-0.054573000
6	3.589219000	1.884243000	-0.673414000
1	1.999905000	3.222287000	-0.337153000
7	4.075398000	0.631461000	-0.654542000
1	4.241461000	2.635522000	-1.098386000
6	5.395267000	0.363757000	-1.202076000
1	5.337949000	-0.410705000	-1.967793000
1	6.082174000	0.046493000	-0.415638000
1	5.775780000	1.275676000	-1.654952000
6	1.423898000	-1.076729000	1.220935000
6	3.901956000	-1.648827000	0.111947000
6	1.989301000	-2.310633000	1.413323000
1	0.490400000	-0.831353000	1.707653000
6	3.220422000	-2.606466000	0.822221000
1	4.877168000	-1.879385000	-0.292345000
1	1.492864000	-3.046813000	2.032759000
1	3.664035000	-3.585691000	0.955326000

Cis-E

6	-0.178741000	-1.774669000	-0.174438000
1	-0.105563000	-2.846289000	-0.329715000
6	-1.481005000	-1.271587000	-0.079635000
6	-1.895141000	0.079499000	-0.391902000
6	-2.524894000	-2.157062000	0.268324000
6	-3.214414000	0.500815000	-0.099946000
6	-1.059011000	0.979419000	-1.072629000
6	-3.779699000	-1.698708000	0.520501000
1	-2.317954000	-3.207661000	0.422291000
6	-3.602197000	1.821329000	-0.365899000
6	-1.460540000	2.257589000	-1.367719000
1	-0.086441000	0.638842000	-1.400724000
1	-4.562331000	-2.363809000	0.860805000
6	-2.731274000	2.687760000	-0.980617000
1	-4.597518000	2.159535000	-0.115282000
1	-0.801390000	2.924104000	-1.909621000
1	-3.050828000	3.700482000	-1.194327000
6	-5.474663000	0.015619000	0.740796000
1	-5.454243000	0.804585000	1.493875000
1	-5.999269000	0.375605000	-0.146090000
1	-6.013181000	-0.837967000	1.144537000
7	-4.123903000	-0.402466000	0.405976000
6	1.058467000	-1.130785000	-0.100499000
6	3.464806000	-0.543839000	-0.292975000
6	2.729738000	0.339087000	0.497715000
6	4.822198000	-0.361283000	-0.501420000
6	3.339564000	1.436599000	1.088145000
6	5.429365000	0.731890000	0.091726000
1	5.390172000	-1.051685000	-1.112256000
6	4.694858000	1.619549000	0.874715000
1	2.775821000	2.137105000	1.690632000
1	6.488423000	0.899251000	-0.058836000
1	5.190473000	2.470993000	1.324105000
16	2.432829000	-1.785570000	-0.918223000
7	1.397030000	-0.037878000	0.616140000
6	0.537337000	0.556076000	1.616961000
1	-0.230536000	-0.165636000	1.887560000
1	1.137247000	0.787337000	2.496605000
1	0.059615000	1.464877000	1.248221000
